# Physiological factors affecting the mechanical performance of peripheral muscles: A perspective for long COVID patients through a systematic literature review

**DOI:** 10.3389/fphys.2022.958333

**Published:** 2022-10-12

**Authors:** Harinivas Rao Suba Rao, Nur Azah Hamzaid, Mohd Yazed Ahmad, Norhamizan Hamzah

**Affiliations:** ^1^ Biomechatronics and Neuroprosthetics Laboratory, Department of Biomedical Engineering, Faculty of Engineering, Universiti Malaya, Kuala Lumpur, Malaysia; ^2^ Department of Biomedical Engineering, Faculty of Engineering, Universiti Malaya, Kuala Lumpur, Malaysia; ^3^ Centre for Applied Biomechanics, Department of Biomedical Engineering, Faculty of Engineering, Universiti Malaya, Kuala Lumpur, Malaysia; ^4^ Clinic for Robotic Rehabilitation, Exercise and Advanced Universiti Malaya Medical Centre, Kuala Lumpur, Malaysia; ^5^ Biosensor and Embedded Systems Laboratory, Department of Biomedical Engineering, Faculty of Engineering, Universiti Malaya, Kuala Lumpur, Malaysia; ^6^ Department of Rehabilitation Medicine, Faculty of Medicine, Universiti Malaya, Kuala Lumpur, Malaysia

**Keywords:** long COVID, skeletal muscles, muscle strength, mechanomyography, physiology

## Abstract

**Background:** Peripheral muscle weakness can be measured quantitatively in long COVID patients. Mechanomyography (MMG) is an alternative tool to measure muscle strength non-invasively.

**Objective:** This literature review aims to provide evidence on the efficacy of MMG in measuring muscle strength for long COVID patients and to determine the physiological factors that may affect the use of MMG in assessing muscle performance.

**Methods:** A systematic literature review was conducted using EBSCO’s MEDLINE Complete. A total of five out of 2,249 potential publications fulfilled the inclusion criteria.

**Results:** The selected studies addressed muscle performance based on the physiological effects of age, gender, and physical activity level. MMG is sensitive in measuring muscle strength for long COVID patients due to its higher signal-to-noise ratio and lightweight accelerometers. Its neglectable skin impedance and low risk of influences during the recording of surface motions make MMG a reliable tool.

**Conclusion:** Muscle performance is affected by age, gender, and physical activity level. Sensors, such as MMG, as well as the length of the muscle and the characteristics of the muscle activity, are important considerations when choosing a sensor for diagnostic evaluation. The efficacy of MMG in measuring muscle strength for long COVID patients and the physiological factors that may affect the use of MMG in assessing muscle performance are discussed.

## Introduction

Long COVID is defined as a post-COVID-19 condition that occurs in individuals with a history of probable or confirmed SARS CoV-2 infection, usually 3 months from the onset of COVID-19, with symptoms that last for at least 2 months and cannot be explained by an alternative diagnosis (WHO, 2022). Common symptoms include deconditioning or fatigue, shortness of breath, cognitive dysfunction, and imbalance (WHO, 2022). Approximately 10% of patients have suffered from these symptoms (Rajan et al., 2021), with fatigue being the predominant feature (87%) (Greenhalgh et al., 2020). Symptoms may be new onset following initial recovery from an acute episode or may persist from the initial illness. Symptoms may also fluctuate or relapse over time (WHO, 2022). This sequelae pattern makes it challenging to identify precisely which individuals will suffer from long-term impairment and the symptom severity (Carfì et al, 2020).

Muscle wasting and weakness are two other significant sequelae following infection (Andrade-Junior et al., 2021). There are, however, several physiological factors that may affect objective muscle performance readings by electromyography (EMG) and mechanomyography (MMG). MMG is a non-invasive method of capturing low-recurrence sidelong motions in dynamic skeletal muscle strands. These motions represent the mechanical companion of EMG-measured engine unit movement. In comparison to EMG, the features of MMG, such as minor skin arrangement, the negligible impact of skin impedance, and the significantly lesser effect of external noise, make this method a viable option to quantify muscle contraction.

Even though EMG has been widely used for muscle evaluation, it has been demonstrated that MMG can evaluate muscle work in a non-intrusive manner, reinforcing its therapeutic potential. In contrast to an EMG signal, the recurring content of a MMG signal reveals important facts on muscle contractile qualities associated with muscle fiber type and organization. The literature delves deeper into the use both of MMG and EMG to depict neuromuscular capacity (Tarata, 2003; Beck et al., 2005).

MMG signals, like EMG signals, are detected using transducers. Mechanical motions or vibrations in muscles are created at a gross level by dimensional changes during isometric or dynamic muscular constrictions. The weight and size of the sensor are important considerations when selecting transducers for MMG. Accelerometers, piezoelectric contact sensors (PECs), and condenser mouthpiece sensors are among the transducers that have been used for MMG estimations, with lightweight accelerometers being recommended because they reduce the risk of unsettling influences during the recording of surface motions (Ibitoye et al., 2014). During both deliberate and animated constrictions, a recorded MMG signal can resemble a muscle’s motor unit actuation mechanism, which is dependent on transient and phantom aspects. MMG root mean square (RMS) and spectral features, such as MMG mean power frequency (MPF), median frequency (MDF), and center frequency (CF), are examples of transitory features that provide data on motor unit enlistment and terminating. At close maximal forces or lengths, these elements can show inverse elements during various kinds of withdrawals, suggesting an end to the enlistment of new motor units (Ibitoye et al., 2016).

The aim of this study is to evince the efficacy of MMG in measuring muscle strength for long COVID patients, and to determine the physiological factors that may affect the use of MMG in assessing muscle performance.

## MMG as a muscle sensor for muscle fatigue assessment relevant in COVID-19 symptom presentation

There are several types of MMG sensors: 1) accelerometer; 2) microphone; 3) piezoelectric; and 4) ultrasonic. Accelerometers are widely used as they are small, compact, cheap, and user-friendly (Ma, 2010). Single-axis (Cè et al., 2013; Talib et al., 2018), double-axis (Tarata, 2003), and tri-hub accelerometer utilization have been reported. Microphones work based on the detection of muscle vibration, whereas phonomyography functions through pressure wave detection produced by muscle vibration. Piezoelectric sensors are the latest addition to the MMG sensor type, whereby a thin strip of piezoelectric material is adhered to the skin surface to quantify the muscle behavior. A condenser microphone gives less signal-to-noise ratio (SNR) in a MMG signal compared to an accelerometer in dynamic contractions.

When muscle contraction occurs, the skin stretches, and muscle belly radius is increased. Muscle activity can then be determined. A trial design of a PEC that contrasted the aftereffects of a PEC with a conventional accelerometer has been reported (Watakabe et al., 1998). An ultrasonic sensor in essence applies density, flexibility, elasticity, and stiffness to measure muscle activity (Tanaka et al., 2003). However, during a muscle fatigue state, the speed of muscle vibro-contraction gradually decreases. Muscle fatigue is quantified as a decrease in maximal strength or power. When this occurs, it will lead to a drop in muscle movement speed. Nonetheless, submaximal contractions can still be maintained (Enoka and Duchateau, 2008).

Mechanically, MMG is superior to other techniques because of the advent features, such as higher sensitivity, lightweight design, and user-friendliness (Pan et al., 2020), progress in signal examination innovation, higher reliability for data compared to other techniques, and the ability to ignore skin impedance (Alves and Chau, 2010). It is also effective in acquiring the low recurrence vibration of muscle action (Orizio et al., 2003). Hence, MMG is the better choice to measure muscle strength in long COVID patients as it has been theoretically demonstrated to have greater sensitivity compared to other alternative sensors or techniques.

MMG innovation is more straightforward to utilize than EMG since it does not need pre-intensification, coupling gel, or direct skin contact. Thus, MMG provides a more viable, more productive, more sterile, and reusable execution. Moreover, the MMG signal is not influenced by any incitement (Shin et al., 2016). It is becoming increasingly accepted as an alternative methodology for clinical applications because of the comfort and accessibility of low-cost accelerometer-based wearables (Plewa et al., 2017). Consequently, MMG has been identified as a reciprocal evaluation to EMG in assessing postural balance and age-related muscle alterations in a variety of kinesiological and clinical examinations (Cè et al., 2015; Hossain et al, 2014).

Additionally, EMG signals are not known to show differences between concentric and eccentric withdrawals (Jaskólska et al., 2004), which lead to severe complications in measuring muscle strength in long COVID patients. MMG is chosen instead of other available sensors, such as EMG, due to its specificity and reliability, which makes it more useful for measuring muscle strength precisely. A MMG sensor has a greater sign sufficiency and SNR compared to an EMG signal. In conducting fatigue tests, MMG sensors have been proven to have greater stability and higher affectability than EMG sensors (Pan et al., 2020). A higher SNR makes the signal quality better and stronger in relation to the noise levels, which allows higher data rates and fewer retransmissions, which offers better throughput in measuring muscle strength for long COVID patients.

However, MMG does have its limitations. Muscle fatigue can affect its parameters, requiring signal processing for compensation (Bowdle et al., 2020; Šimunič et al., 2015). A natural multi-layered muscle arrangement also contributes to decreased sensitivity. Certain anatomical conditions, such as limb amputation or any physical deformity, make MMG sensors less suitable (Ma, 2010).

## Methodology

### Study objectives

The systematic review objectives were: 1) to provide evidence on the efficacy of MMG in measuring muscle strength for patients with COVID-19 infection; and 2) to determine the physiological factors that may affect the use and interpretation of MMG signals in assessing muscle performance following COVID-19 infection.

### Review search strategy

A total of 2,249 peer-reviewed citations were found through searches performed on EBSCO’s MEDLINE Complete. Relevant citations were obtained between 2019 and 2022 by using the following specific words in the “Article titles” search field: “long COVID” or “post-COVID” and “peripheral muscle.” The citations were further refined by limiting them to the English language, for humans only, and journals available with full text and abstract. Following this, further citations were excluded due to irrelevance to the topic. All titles were manually screened to ensure that they were relevant to peripheral muscles. A comprehensive initial search included journal articles, review articles, conference papers and proceedings, book chapters, and reports.

### Eligibility criteria

The electronic search was made with the central focus on the “effects on long COVID patients” and “alterations in the patients’ muscle strength.” Further derivations included gender, age, and physical activity. Studies were excluded if non-human subjects were used and if non-human subjects’ data were presented.

### Data extraction

Data were extracted for searches on: 1) the conditions of long COVID; or 2) the assessments and effects of muscle strength. Information inferred from each article included study topic, study objectives, stimulation protocol, physiological factors, the methodology adopted, results presentation, study outcomes, and suggestions for future studies.

### Validity assessment

All authors were involved in the extraction of data from the identified citations to reduce the risk of bias. Several studies adopted different types of assessments for muscle strength.

## Results

### Included studies

**FIGURE 1:**
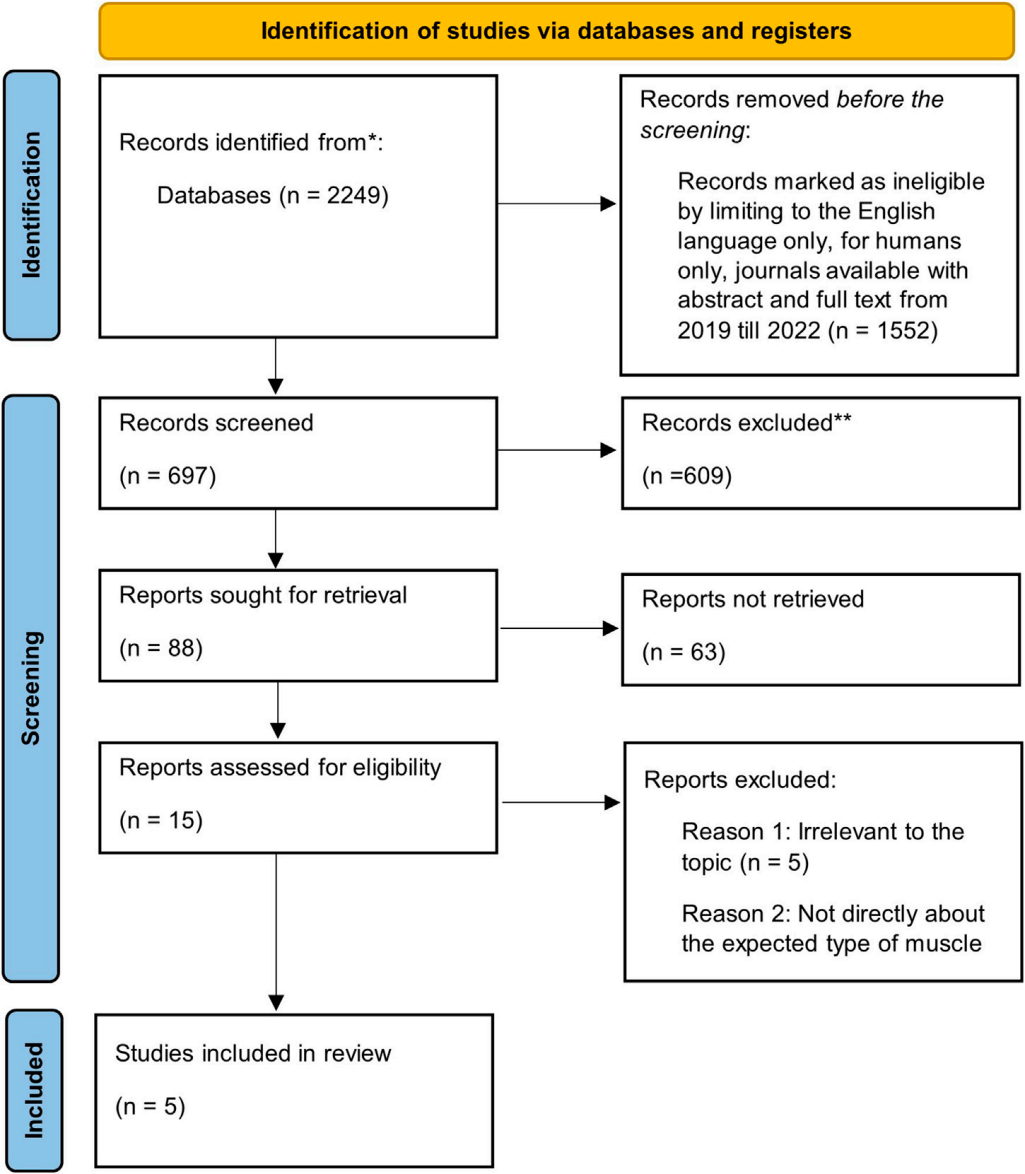
The PRISMA flowchart for article selection.

Out of the 2,249 citations retrieved, only five satisfied the inclusion criteria (Figure 1). Five of the studies reported at least one of the following outcomes: skeletal muscle alterations; short-term muscle loss; loss of muscle strength; physiological factors such as sarcopenia (a type of muscle disorder); physical activity; mood; and sleep quality. The main objective of this paper is to address: 1) the effects on skeletal muscle strength; and 2) the physiological factors affecting muscle strength.

Age, physical activity, and gender were identified as the physiological factors that can affect muscle strength measurement *via* MMG (Table 1).

**TABLE 1:** Summary of selected post-COVID-19 studies.

Study/title	Study population	Study objective	Study outcomes	Physiological factors
Gérard et al. (2021). Long-term evolution of malnutrition and loss of muscle strength after COVID-19: A major and neglected component of long COVID-19	Post-acute COVID-19 patients *n* = 136 (of total 549 patients, 132 died, 288 discharged home)	To investigate persistent side effects, nourishment status, the development of muscle strength, and performance status (PS) 6 months post-discharge in a cohort of COVID-19 survivors.	Obese subjects, as well as patients who have remained in intensive care, have a higher risk of functional loss or undernutrition 6 months after a severe COVID-19 infection. Malnutrition and loss of muscle strength ought to be considered in the clinical appraisal of these patients.	Physical activity
Gobbi et al. (2021). Skeletal muscle mass, sarcopenia and rehabilitation outcomes in post-acute COVID-19 patients	Post-acute COVID-19 patients *n* = 34	To explore the effect of the presence of sarcopenia upon admission to a post-intense COVID-19 patient rehabilitation unit on body arrangement and functional and respiratory capacity at discharge.	Upon admission, the predominance of sarcopenia among the patients was 58%. In every one of the 34 patients, the pattern of progress in all the respiratory, body composition, muscle strength, and practical boundaries was considered. Observing muscle mass and strength in post-intense COVID-19 patients gives an impression of being a vital indicator of rehabilitation outcomes.	Age
Kirwan et al. (2020). Sarcopenia during COVID-19 lockdown restrictions: Long-term health effects of short-term muscle loss	Post-acute COVID-19 patients *n* = 118	To investigate the mechanisms of sarcopenia and their relation to the present information on the impacts of COVID-19 confinement on physical activity, dietary habits, sleep, and stress, as well as prolonged bed rest because of COVID-19 hospitalization.	Reductions in physical activity, disruption to normal eating habits, stress, and altered sleeping patterns will put older people at greater risk of sarcopenia, which, along with its own implications for quality of life and mobility, can lead to easier COVID-19 infection. Physical activity will assume a vital role for rehabilitation.	Age and physical activity
Soares et al. (2022). Skeletal muscle alterations in patients with acute COVID-19 and post-acute sequelae of COVID-19	Post-acute COVID-19 patients *n* = 41 (40–88 years old)	To explore skeletal muscle alterations’ risk factors and confounding factors in acute COVID-19 and post-acute sequelae of COVID-19 (PASC) patients.	Patients with serious COVID-19 and PASC patients experience the adverse effects of skeletal muscle weakness and dyspnea, as well as fatigue, each seen in approximately 53%–63% of patients, being more common in women and patients with a higher disease severity of acute COVID-19.	Gender
Tanriverdi et al. (2021). Extrapulmonary features of post-COVID-19 patients: Muscle function, physical activity, mood, and sleep quality	Post-acute COVID-19 patients *n* = 48 (39.2 ± 7.9 years, 54.2% women)	To explore extrapulmonary highlights in post-COVID-19 patients who recuperated from mild and moderate disease severity in the mid-term. Physical activity level (PAL), mood, and sleep quality were assessed using the International Physical Activity Questionnaire, Hospital Anxiety and Depression Scale, and Pittsburgh Sleep Quality Index.	Handgrip and quadriceps weakness was monitored in 39.6% and 35.4% of the participants, respectively; the PAL was low in 39.6%, moderate in 33.3%, and high in 27.1% of the participants. Anxiety, depression, and poor sleep quality were observed in 33.3%, 29.2%, and 50% of the participants, respectively.	Physical activity

*Notes:* After initially selecting relevant citations, some were excluded based on the following inclusion criteria: English language only; for humans only; and journals available with abstract and full text from 2019 to 2022. Other citations were excluded due to irrelevance to the topic or for not being related to physiological factors.

## Physiological factors that may affect MMG reading in COVID-19 patients

### Age

A person’s age is highly associated with MMG quantification. The younger age group has higher muscle strength. The peak torsion is higher (by 50%) between the ages of 11 and 15 when compared to between the age of 12 to 14. Between the ages of 14 and 15, the knee flexor/extensor ratios decrease significantly. For all angular velocities, the pubescence period is the most vital determinant of the peak torque level (based on age, size, and height) (*p* = 0.0001). Tanner staging, otherwise known as sexual maturity rating (SMR), is an objective characterization framework that providers use to document and track the development and sequence of secondary sex attributes of children during pubescence (Emmanuel and Bokor, 2017). There is total of five stages. Between Tanner stages 1 and 5, muscle strength increases dramatically, with the biggest improvement occurring between stages 2 and 4 (Degache et al., 2010). It has been established that as a person ages, the degree of strength of each muscle mass deteriorates. This deterioration is linked to an increased risk of falls, hip fractures, and bone mineral density loss (Lindle et al., 1997). Strength improvement from pre-adolescence to adolescence is presumably influenced by neuromuscular adaptations through biological maturity (Gillen et al, 2019).

Age, morphology, muscle abilities, and the duration at which the muscle is tested predict the quantity of pressure that may be generated (Lindle et al., 1997). Age-associated variations of the neuromuscular system complicate the joint-perspective-EMG and joint-perspective-MMG relationships. There is progressive reduction of fast-twitch motor devices and, concurrently, re-innervation of deserted fast-twitch muscle fibers with progressive age. This may change the pressure-EMG and pressure-MMG relationships. Type II muscle fibers are also lower in older individuals and this physiological change causes a decrease in EMG and MMG amplitude. In addition, different muscles have a different morphology that changes with age.

Sarcopenia is an age-related decrease of muscle mass and strength. It is a significant antecedent of frailty, impairment, and disability (Fielding et al., 2011; Zhang et al., 2018). Patients with sarcopenia (portrayed by reduced muscle mass and muscle strength) have poor immune response and metabolic pressure during acute infection. Patients with sarcopenia are most likely to have worsened sequelae following a more severe category of COVID-19 infection with poorer prognosis (Wang et al, 2021). Evidence has also reported that patients are in a much worse category in the presence of sarcopenia and a lower oxygen saturation of ≤93% (i.e., a severe category of COVID-19 infection) (Gobbi et al., 2021; Karahan and Katkat, 2021). In the older age group, reduction in physical activity, changes in dietary intake/patterns, high stress levels, and altered sleeping patterns are risk factors for sarcopenia. These factors are also associated with poor quality of life and impaired mobility, both adding to poor outcomes following COVID-19 infection (Kirwan et al., 2020). Therefore, age (and its physiological attributes) is most likely an independent physiological factor for poor outcomes following COVID-19 infection.

### Gender

Gender is an important physiological element that affects muscle strength. One study reported that a man’s maximum force creation would be reduced more noticeably than a woman’s following COVID-19 infection (Hill et al., 2018). Androgens’ anabolic effects on the skeletal muscles of males increases muscle bulk and thus muscle strength (Kline and De Luca, 2016). However, less is known on the effects of estrogen on female skeletal muscle mass (Hill et al., 2018). Interestingly, the severity and mortality of COVID-19 are higher in males than females. The male gender is a known risk for COVID-19 infection and is viewed as more vulnerable when related to endurance and infection control (Costeira et al., 2021).

Patients with severe COVID-19 infection [post-acute sequelae of COVID-19 (PASC) patients] experience pronounced fatigue, muscle weakness, and shortness of breath. Approximately 53–63% of patients with a severe category of COVID-19 infection are women (Soares et al., 2022). However, a study has shown that men with COVID-19 infection are more likely to die from the infection or to develop a severe infection category that requires intensive care unit (ICU) and ventilatory support. Those who recovered following infection suffered from prolonged residual symptoms (Ya’qoub et al, 2021). The male gender is an independent risk factor for ICU care following COVID-19 infection. Other factors for the same gender include obesity, persistent kidney illness, and hypertension. Men are also more likely to suffer from prolonged post-COVID-19-related symptoms of fatigue and shortness of breath (Maestre-Muñiz et al., 2021).

Female skeletal muscle strength is affected by the level of serum estrogen, specifically on the mass and type of contractile proteins. It has also been discovered that progesterone itself enhances the basal rate of the muscle protein blend (Orizio et al., 2003). The basal rates of muscle protein synthesis have been found to be 20–30% higher in menopausal women than in younger aged or premenopausal women, which indicates that estrogen deficiency may improve the rate of protein synthesis (Robergs et al., 2004). Uniquely, estrogen may have a protective effect for women against COVID-19 infection. As women age to reach the menopausal state (and hence reduced serum estrogen levels), this results in a physiological muscle mass reduction. Deconditioning that may occur in the severe COVID-19 infection category will lead to a longer recovery process following the infection (Costeira et al., 2021). Hence, the severity of COVID-19 sequelae may vary at different physiological stages of a woman’s life.

### Level of physical activity

The intensity of regular activity has an impact on muscle size and strength, especially resistance training, which can increase the strength of lean muscles post-COVID-19 infection (Adelnia et al., 2019). Obesity and patients with prolonged ICU care have a higher risk of functional loss and malnutrition lasting for 6 months after a severe COVID-19 infection. Malnutrition and loss of muscle strength/mass status ought to be addressed in these patients (Gérard et al., 2021) as a high level of physical activity is associated with the reduced severity of COVID-19 infection.

Active muscles produce chemicals that can improve immune functioning, which in turn reduces the extent of the infection and inflammation. COVID-19 infection causes a systemic inflammatory reaction that primarily leads to severe acute respiratory distress syndrome (ARDS). Physical activity is a powerful preventative and therapeutic intervention not only for COVID-19 infection but also for common pre-existing chronic conditions that increase risk of the viral infection (Jordan et al, 2020)*.*

Additionally, the benefits of physical activity encompass psychological well-being through the prevention and treatment of anxiety and depression directly caused by COVID-19 infection or because of the stressful global crisis entailed by the pandemic (Powell et al., 2018). Therefore, it can be said that physically stronger people or physically active people get COVID with less severity and that they are more resistant to fatigue (Sallis et al, 2020).

### Muscle strength assessment

A study had reported that patients in the severe COVID-19 infection category had a decrease in the cross-sectional rectus femoris area and the thickness of the anterior quadriceps muscle (rectus femoris and vastus intermedius) with increased echogenicity detected between days 1–10 of infection (Andrade-Junior et al., 2021). These findings could shed light on skeletal muscle atrophy and functioning in COVID-19 patients. MMG can be applied to assess specific portions of the muscle fiber structure instead of the biopsy technique. The length of the peripheral nervous system (PNS) excitement, which impacts the electrical strength added to the muscle’s tissues, can impact the abundancy and timing of the MMG waveforms generated from a contracting muscle (Jaskólska et al, 2003).

One study investigated individuals’ dietary status, muscle strength changes, and performance status among those with COVID-19 persistent symptoms. Out of 119 cases, i.e. 91% of 136 post-COVID patients who were still weak and undergone 6 months intervention, post dicharge, 36% still had persistent malnutrition and 14.3% complained of substantial loss of muscle power. Obesity was shown to be more common in those with impairment than those without, with a higher proportion being treated in the ICU. Psychiatric co-morbidities (mood disorders, anxiety, or post-traumatic stress syndrome) were found in 10% of individuals with long COVID, whereas 7.6% had extended pneumological symptoms and 4.2% had neurological problems. In summary, obese individuals managed in the ICU were more likely to experience functional loss following a severe COVID-19 infection (Gérard et al., 2021).

In addition to the physiological factors that potentially affect MMG performance, it has also been reported that correlations between torque and mechanomyogram in short, evoked contractions are strongly linear (Ibitoye et al., 2016). However, muscle fatigue will have an effect on the time the muscle takes to recover in a longer test session. Sensors, such as piezoelectric and muscle contraction sensors, as well as the length of the muscle and the type of muscle action, are critical factors in monitoring specific muscle status (Uwamahoro et al, 2021; Siddiq et al., 2020)

Typically, the qualities of a reliable MMG transducer include the following: 1) extreme affectability within the muscle vibrational recurrence range, i.e., 2–100 Hz, and extreme affectability to arbitrary markers (clamor); 2) sensor connection simplicity and normalization; 3) biocompatibility and relevance in a logical climate; and 4) cost adequacy when compared to various logical assessment methods.

There is inter-character changeability that may have negative impacts, such as differences in weight, muscle length, and tissue thickness among the sensors. Standardization is essential because the MMG signal is variable and influences numerous components that require regulation, and each of these components shift from one situation to the next. Similarly, the MMG estimations might be collected inside the fundamental devices of vibration from the skeletal muscle tissues, measured using m/s^2^, making standardization unnecessary (Shin et al., 2016). This is a consideration when the affectability of sensors is frequently expressed in mV/g, where g is the speed increase considering gravity when an accelerometer sensor is used instead of the m/s^2^ unit.

## Conclusion

Age, gender, and the level of physical activity are the main physiological factors that can influence MMG readings of muscle strength. The efficacy of MMG in measuring muscle strength for long COVID patients and the physiological factors that may affect the use of MMG assessing muscle performance have been discussed in this paper. Future studies can focus on these factors when measuring muscle strength for long COVID patients using MMG. MMG has been theoretically proven to be capable of measuring muscle strength more efficiently compared to other alternative or current techniques.

## Data Availability

Publicly available datasets were analyzed in this study. This data can be found here: Web of Science; Scopus; and EBSCO’s MEDLINE Complete.
